# Increased Anti-Phospholipid Antibodies in Autism Spectrum Disorders

**DOI:** 10.1155/2013/935608

**Published:** 2013-09-23

**Authors:** Milo Careaga, Robin L. Hansen, Irva Hertz-Piccotto, Judy Van de Water, Paul Ashwood

**Affiliations:** ^1^Department of Medical Microbiology and Immunology, University of California, Davis, USA; ^2^MIND Institute, University of California, 2805 50th Street Sacramento, Davis, CA 95817, USA; ^3^Department of Pediatrics, University of California, Davis, USA; ^4^Department of Public Health Sciences, Division of Epidemiology, University of California, Davis, USA; ^5^Division of Rheumatology, Allergy and Clinical Immunology, University of California, Davis, CA, USA

## Abstract

Autism spectrum disorders (ASD) are characterized by impairments in communication, social interactions, and repetitive behaviors. While the etiology of ASD is complex and likely involves the interplay of genetic and environmental factors, growing evidence suggests that immune dysfunction and the presence of autoimmune responses including autoantibodies may play a role in ASD. Anti-phospholipid antibodies are believed to occur from both genetic and environmental factors and have been linked to a number of neuropsychiatric symptoms such as cognitive impairments, anxiety, and repetitive behaviors. In the current study, we investigated whether there were elevated levels of anti-phospholipid antibodies in a cross-sectional analysis of plasma of young children with ASD compared to age-matched typically developing (TD) controls and children with developmental delays (DD) other than ASD. We found that levels of anti-cardiolipin, **β**2-glycoprotein 1, and anti-phosphoserine antibodies were elevated in children with ASD compared with age-matched TD and DD controls. Further, the increase in antibody levels was associated with more impaired behaviors reported by parents. This study provides the first evidence for elevated production of anti-phospholipid antibodies in young children with ASD and provides a unique avenue for future research into determining possible pathogenic mechanisms that may underlie some cases of ASD.

## 1. Introduction

Autism spectrum disorders (ASD) are a group of neurodevelopmental disorders characterized by stereotyped interests, repetitive behaviors, and impairments in communication and social interaction. Currently 1 in 88 children has been identified as having ASD [1]. Despite the high incidence of ASD, etiology and pathogenesis remain poorly understood. Current research suggests a significant role for immunodysregulation and autoimmune processes in the pathophysiology of ASD, with the existence of autoantibodies directed against neuronal proteins repeatedly demonstrable in a substantial number of children with ASD (reviewed in [2, 3]).

The reported targets of autoantibodies exhibited by children and adults with ASD are diverse and range from neurotransmitter receptors such as serotonin receptors, markers of astroglial activation such as glial fibrillary acidic protein (GFAP), and myelin sheath cellular products such as myelin basic protein (MBP), as well as yet unidentified neuronal protein targets (reviewed in [4]). Moreover, the presence of neuronal protein-specific autoantibodies are associated with increased behavioral impairments and more severity in children with ASD [5], suggesting a link between the autoimmune processes and behavioral dysfunction. For example, autoantibodies directed against a 45 kDa protein present in the cerebellum were not only found more frequently in children with ASD but were also associated with lower adaptive and cognitive function, as well as increased aberrant behaviors [6, 7]. However, replication studies of antibody-specific antigen targets, such as MBP and GFAP, have been inconsistent, suggesting that further studies are needed to identify the target or targets and/or associated autoimmune phenomena [8, 9]. Recent studies have highlighted a role for anti-phospholipid antibodies in altering function and behaviors such as cognition, anxiety, and hyperactivity. 

Anti-phospholipid antibodies recognize a number of diverse targets including cardiolipin, phosphoserine, and *β*2-glycoprotein 1. They are found in roughly ten percent of the population [10, 11] but are thought to cause pathology in only a small segment of those with antibodies. Elevated levels of anti-phospholipid antibodies have been found in the blood and cerebral spinal fluid of psychiatric patients having hallucinatory and/or delusionary episodes [12]. In individuals with neuropsychiatric systemic lupus erythematosus, elevated titers of anti-cardiolipin antibodies are reported most often in patients with cognitive impairment, psychosis, depression, seizures, chorea, and migraines [13]. Moreover, in animal models, administration of anti-phospholipid antibodies to rodents can induce a number of psychological side effects, including increased anxiety and decreased cognition learning and memory [14, 15]. 

When anti-phospholipid antibodies are present in an individual consistently over time, along with arterial or venous thrombosis or pregnancy morbidity, this is termed antiphospholipid syndrome (APS). Although rare in children, APS does occur and is thought to be underdiagnosed likely due to lack of testing in this population, as these autoantibodies are generally related to fertility and cardiovascular events [16]. It is still unclear what the presence of elevated levels of anti-phospholipid antibodies signify in the pediatric population in terms of comorbidities [17].

Based on the links between anti-phospholipid antibodies and altered behaviors and cognition, we evaluated a panel of autoantibodies associated with APS including anticardiolipin, antiphosphoserine, and anti-*β*2-glycoprotein 1 in plasma from a large cohort of well-characterized children enrolled in a population-based case-control study. To better define the immune status of children with ASD, autoantibody profiles were assessed in children 24–82 months of age who had ASD and compared with typically developing children and children with developmental delays other than ASD who were of the same age and lived in the same geographical area. In addition, antibody levels were investigated for associations with clinical behavioral outcomes.

## 2. Methods

### 2.1. Subjects

One hundred and nine participants who were enrolled through the population-based case controlled Childhood Autism Risks from Genetics and Environment (CHARGE) study conducted at UC Davis MIND Institute were recruited to this study [18]. The study protocols including recruitment and behavioral assessments have been described in detail [18–20]. In brief, following clinical evaluation for diagnostic confirmation, participants were placed in one of three groups: (1) children with a confirmed diagnosis of ASD [*N* = 54, median age 44.8 months (interquartile range 32.0–57.7), 45 males]; (2) children diagnosed with developmental delay but not ASD [*N* = 22. median age 41.7 months (IQR 25.7–57.8), 18 males.]; or (3) children who were confirmed as typically developing controls [*N* = 33, median age 40.6 months (IQR 27.7–53.6), 27 males]. Final diagnosis of ASD was confirmed by the Autism Diagnostic Interview-Revised (ADI-R) [21] and the Autism Diagnostic Observation Schedule (ADOS) [22]. The ADOS and ADI-R consist of a standardized, semistructured interview and a diagnostic algorithm from the Diagnostic and Statistical Manual of Mental Disorders, Fourth Edition Text Revision (DSM-IVTR) [23], with definitions of autism from the International Classification of Diseases, Tenth Revision (ICD-10) [24]. The administration of all diagnostic instruments was carried out by experienced clinicians at the UC Davis MIND Institute.

Additional behavior testing included the Aberrant Behavior Checklist (ABC), Mullen Scales of Early Learning (MSEL), and Vineland Adaptive Behavior Scales (VABS). The ABC was taken by parents of children in the study and consists of questions designed to measure the severity of autism-associated behaviors, including irritability, lethargy, stereotypy, hyperactivity, and inappropriate speech. Assessment scores for the ABC range from 0 to 174, with higher scores indicating more severely affected behavior. In addition to the ABC, children enrolled in the study were assessed for cognitive function using MSEL. The MSEL has components for visual reception, fine motor, receptive language, and expressive language, each of which yields a *T* score with mean = 50 and SD = 10. Adaptive function was assessed through parental interview using the VABS. The VABS has components for communication, daily living, socialization, and motor skills. These components each component yields a score from 20 to 160 with a mean among typically developing children of 100.

Participants did not differ for age or sex ratios. All children were medication-free and in good health and without diagnosis of autoimmune conditions at time of the blood draw. This study was approved by the institutional review boards in the University of California, Davis. Informed consent was obtained prior to participation.

### 2.2. Antibody Analysis

For each subject peripheral blood was collected in acid-citrate-dextrose Vacutainers (BD Biosciences; San Jose, CA), centrifuged at 2300 rpm for 10 min and the plasma harvested. Plasma was aliquoted and stored at −80°C until antibody levels were measured. The IgG antibody levels of anticardiolipin, antiphosphoserine, and anti-*β*2-glycoprotein 1 were assessed by commercial ELISA (Orgentec, Mainz, Germany) using the manufactures protocol. In brief, plasma samples were diluted 1 : 100 in assay buffer, and 100 uL of diluted plasma was loaded on plate in duplicate along with calibrators and controls. Samples were incubated for 30 minutes at room temperature. Samples were visualized using 3,3′,5,5′-tetramethylbenzidine (TMB) substrate and read at 450 nm. All results are reported in IgG phospholipid units per milliliter (GPL-U/mL).

### 2.3. Statistical Analysis

Data analysis was performed using STATA 12 software. Data was determined as nonparametric using Shapiro-Wilks test for normality. Wilcoxon rank-sum tests were used to compare antibody levels between subject groups. Spearman correlations were used to determine the association between anti-phospholipid antibodies and variations in scores on behavioral, cognitive, and adaptive assessments. A probability value (*P*) of less than 0.05 was considered to be significant.

## 3. Results

### 3.1. Anti-Phospholipid Antibody Levels in ASD

**Figure 1:**
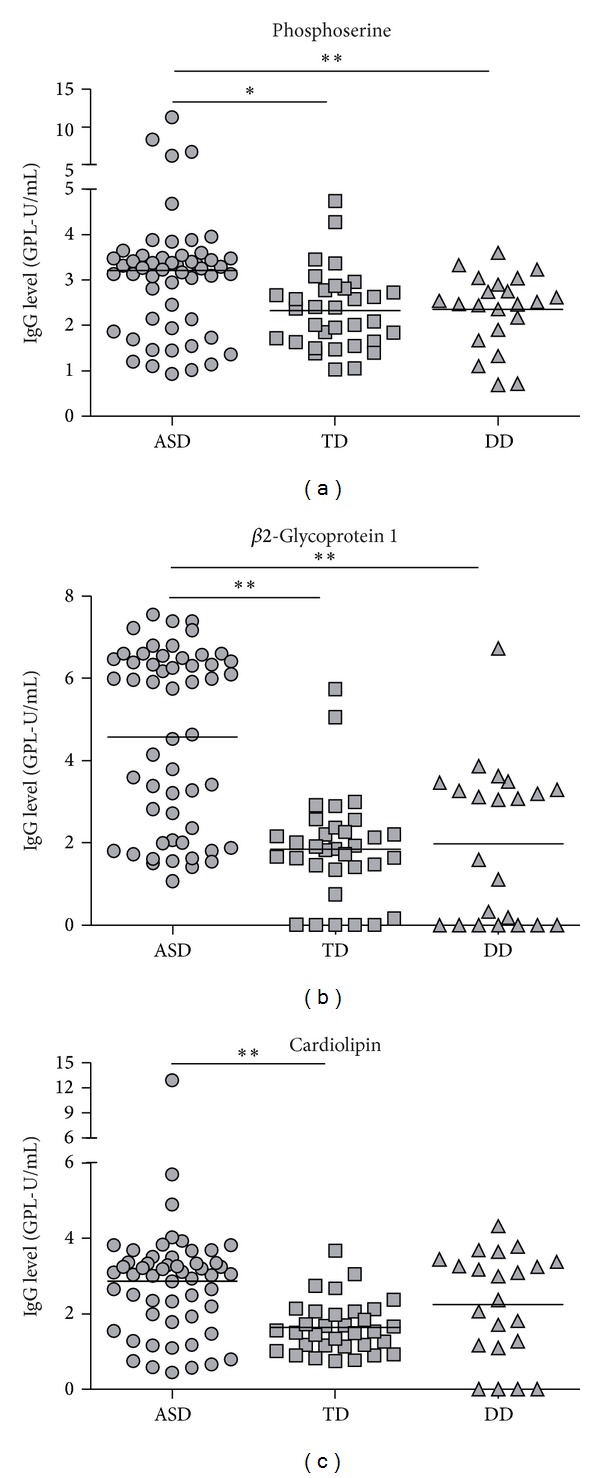
Anti-phospholipid antibody levels. (a) ASD subjects were found to have significant (*P* < 0.01) levels of anti-phosphoserine and (b) anti-*β*2-glycoprotein 1 antibodies compared with TD and DD controls. Levels of (c) anti-cardiolipin were found to be significantly (*P* < 0.001) higher in ASD compared with TD control, but differences to DD control did not reach significance. *P < 0.01, and **P < 0.001.

An approximate 38% increase in anti-phosphoserine antibody levels were observed in children with ASD compared with TD controls (mean 3.209 ± SEM 0.204 versus mean 2.324 ± SEM 0.149; *P* < 0.01) and a 37% increase compared with children with DD (mean 3.209 ± SEM 0.238 versus mean 2.344 ± SEM 0.172; *P* < 0.01) (Figure 1). There was also a 149% increase in anti-*β*2-glycoprotein 1 antibody levels in children with ASD compared with age-matched TD controls (mean 4.584 ± SEM 0.294 versus mean 1.845 ± SEM 0.224; *P* < 0.001) and a 132% increase over children with DD (mean 4.584 ± SEM 0.294 versus mean 1.975 ± SEM 0.406; *P* < 0.001). Antibody levels of anticardiolipin were increased approximately 75% higher in children with ASD compared with TD controls (mean 2.873 ± SEM 0.245 versus mean 1.642 ± SEM 0.121; *P* < 0.001), and there was a trend toward elevated levels in children with ASD compared with DD controls, although this did not reach statistical significance after multiple comparison correction (Figure 1). 

### 3.2. Association of Anti-Phospholipid Antibody Levels and Behaviors

We next examined whether anti-phospholipid antibody levels were associated with impairments in behavior. Significant associations were found between all three anti-phospholipid antibodies assessed and increased severity of behaviors, such as lethargy, irritability, and stereotypic behaviors as assessed by the ABC. Impairments in cognitive and adaptive behaviors as measured by MSEL and VABS were also associated with increased antibody levels. These impairments included deficits in functional communication on the VABS and receptive and expressive language domains measured by the MSEL **(** Table 1 **)**. Although there were strong correlations observed in the pediatric population as a whole, there were no significant differences found when analyzing within the individual groups based on diagnosis. 

## 4. Discussion

In this study we demonstrate that the levels of anti-phospholipid antibodies in children with ASD are significantly elevated when compared with typically developing children and children with developmental delays other than ASD. Furthermore, elevated anti-phospholipid antibodies are associated with increased impairments in a number of clinical cognitive and behavioral measures such as stereotypy, hyperactivity, and communication. Together with previous studies in the field demonstrating the increased presence of autoantibodies in children with ASD, this study adds further support for a possible role for autoimmune phenomena in the pathogenesis of ASD [25]. Several previous studies have shown that increased anti-phospholipid antibodies are present in a number of neuropsychiatric conditions; however, it is currently unclear what, if any, pathologic significance these anti-phospholipid antibodies have in behavioral disorders including ASD. In addition, it is noteworthy that while this study found elevated anti-phospholipid antibodies in children with ASD, the levels are below what is considered clinically significant levels for APS. These current data highlight the importance for further research to investigate the role of anti-phospholipid antibodies in a variety of childhood behavioral disorders.

 APS in children is thought to be rare; however, current assessments are biased away from recognizing the syndrome in prepubescent individuals [17]. One study demonstrated that anti-phospholipid autoantibody levels are elevated in a significant number of children, suggesting that elevated levels of anti-phospholipid antibodies may have importance in children even in the absence of the defining clinical features of APS, such as arterial or venous thrombosis or pregnancy morbidity that is observed in adults [26]. The additional symptoms of APS vary greatly in adults, with cognitive [27], neuropsychiatric [12, 13], and neuromotor [28] symptoms having been observed. Animal models of APS, in which antibodies isolated from individuals with APS are transferred into mice, demonstrate that the effects of the antibodies are associated with neuropsychiatric symptoms such as anxiety, hyperactivity, and impairments in cognition [15, 29, 30]. 

Anti-phospholipid antibodies have been associated with numerous central nervous system involvements, with many symptoms such as stroke and optic neuropathy thought to result from thrombic events. However, the exact mechanism of how these antibodies cause pathology is unknown (reviewed in [31, 32]). It is unlikely that the thrombotic events are responsible for all APS-related symptoms; in many subjects with chorea, lesions are not apparent on CT scans, suggesting that a more direct cellular mechanism could be involved [25, 33, 34]. Direct binding of anti-phospholipid antibodies to neurons has been demonstrated in human neurons *ex vivo,* and these antibodies have been shown to permeabilize and depolarize brain synaptoneurosomes [35, 36]. In mice, when anti-phospholipid antibodies derived from human subjects are administered they recognize neuronal targets and have been shown to decrease astroglia proliferation [37]. It is not clear how this could translate to pathology in ASD, but many studies have shown the increased presence of autoantibodies that interact with neuronal targets. The exact targets are generally not known in ASD, but it is possible that these antibodies, or at least a fraction, could be anti-phospholipid antibodies [7, 25, 34]. In addition, anti-phospholipids may be related to other previous findings such as MBP-specific antibodies [5]. In subjects with SLE increased, elevated anti-cardiolipin antibodies titers are associated with increased myelin binding antibodies [38].

Alternatively, anti-phospholipid antibodies may represent a biomarker for nonspecific neuronal damage or inflammation. In a study looking at anti-neuronal antibodies in 129 young children with and without ASD, 43% of children showed some positive staining for brain reactive antibodies [38]. Although a differential pattern of staining was not readily apparent between those children with ASD compared with controls, those children who did show positive autoantibody staining displayed more severe score on the Child Behavior Checklist (CBCL) [39]. This suggests that non-specific anti-brain antibodies may hearken a more general developmental impairment. In fact, in ASD numerous antibodies directed against brain or central nervous system tissue have been identified (reviewed in [2]). The targets of these antibodies are quite diverse and include serotonin receptors [40], MBP [41], nucleus [42], and GFAP [43], as well as numerous unidentified protein targets. Moreover, unique autoantibody targets seem to be found only in subsets of children with ASD, and their detection has been difficult to replicate across studies; primary examples are antibodies against such targets as MBP and GFAP [8, 9]. Additionally, anti-phospholipid antibodies have been associated with a number of infectious agents, such as syphilis or HIV [44, 45]. However, there is no evidence of increased rates of infection in children with ASD at the ages reported in this study, and all participants in the study were screened for illness at the time of the blood draw [46, 47]. Given the apparent ability of anti-phospholipid antibodies to discriminate children with ASD compared with controls as seen in this study, these antibodies may offer additional novel biomarkers for evaluating pathogenic mechanisms and possible targeted treatments for children with ASD. 

While this study is limited due to its cross-sectional nature and further longitudinal testing is warranted, it is among the first to attempt to measure anti-phospholipid antibodies in a pediatric population with ASD with age-matched controls. The associations between anti-phospholipid antibody levels and impairments in behaviors may be of significance to young children beyond those with ASD. In particular, we demonstrate that these autoantibodies are associated with impairments in behaviors similar to previous studies looking at unidentified neuronal targets [7]. These observations warrant further study. 

## 5. Conclusion

In summary, the findings of increased anti-phospholipid antibody levels in young children with ASD, and the association between antibody levels and impaired behaviors in the pediatric population as a whole, offer potential new targets for understanding the mechanisms involved in the pathogenicity of ASD. Our novel preliminary findings support the importance of further study of the biological impact of autoantibodies and their association with behavioral and cognitive impairments in children with ASD.

## Figures and Tables

**Table 1:** Association analysis of anticardiolipin, *β*2-glycoprotein 1, and antiphosphoserine with behavioral outcome measures as assessed by the Aberrant Behavior Checklist (ABC), Mullen Scales of Early Learning (MSEL), and Vineland Adaptive Behavior Scales (VABS) using Spearman's rank correlations demonstrated that there were significant correlations between anti-phospholipid antibody levels and the severity of impairments in behavior in participants enrolled in this study. For the ABC, a higher score corresponds to more behavioral impairments. For the MSEL and VABS, a lower score corresponds to increased cognitive and adaptive impairments.

Autoantibody profile	Cardiolipin		*β*2-Glycoprotein 1		Phosphoserine	
	*P* value	*r*	*P* value	*r*	*P* value	*r*
Aberrant Behavior Checklist						
Subscale I: irritability	<0.001	0.368	0.001	0.343	0.002	0.312
Subscale II: lethargy	0.001	0.334	<0.001	0.406	0.001	0.317
Subscale III: stereotypy	0.002	0.309	<0.001	0.421	0.002	0.309
Subscale IV: hyperactivity	0.010	0.257	0.001	0.343	0.040	0.206
Subscale V: inappropriate speech	0.409	0.084	0.010	0.258	0.865	0.017
Subscale VI: moods	<0.001	0.345	<0.001	0.346	0.002	0.303
Mullen Scales of Early Learning						
Visual reception	0.031	−0.206	0.002	−0.291	0.042	−0.195
Fine motor	0.004	−0.272	0.002	−0.292	0.002	−0.300
Receptive language	0.009	−0.249	<0.001	−0.356	0.003	−0.286
Expressive language	0.004	−0.271	<0.001	−0.350	0.007	−0.257
Vineland Adaptive Behavior Scales						
Communication	0.011	−0.244	0.003	−0.284	0.001	−0.324
Daily living skills	0.047	−0.190	0.007	−0.255	0.014	−0.234
Socialization	0.022	−0.219	0.002	−0.289	0.002	−0.287
Motor skills	0.088	−0.164	0.127	−0.147	0.014	−0.236
